# Image of the Month: How to Select the Ideal Surgical Approach in Male Anorectal Malformation with No Visible Fistula

**DOI:** 10.1055/s-0040-1721042

**Published:** 2020-11-23

**Authors:** Anisha Apte, Elise McKenna, Marc A. Levitt

**Affiliations:** 1 Department of Surgery, The George Washington University School of Medicine and Health Sciences, Washington, District of Columbia, United States; 2 Department of General and Thoracic Surgery, Children's National Medical Center, Washington, District of Columbia, United States; 3 Division of Colorectal and Pelvic Reconstruction Surgery, Children's National Medical Center, Washington, District of Columbia, United States

**Keywords:** anorectal malformation, distal colostogram, anorectoplasty

## Abstract

We present a case of a 6-month-old male infant with an anorectal malformation (ARM) who underwent colostomy as a newborn, and now presents for definitive repair. A colostogram is shown to identify the malformation and to help plan for the ideal surgical approach. The case is presented with a focus on surgical strategies for management of ARM in the male infant, with questions for the readers posed in a quiz format.

## Case Report

**Fig. 1:**
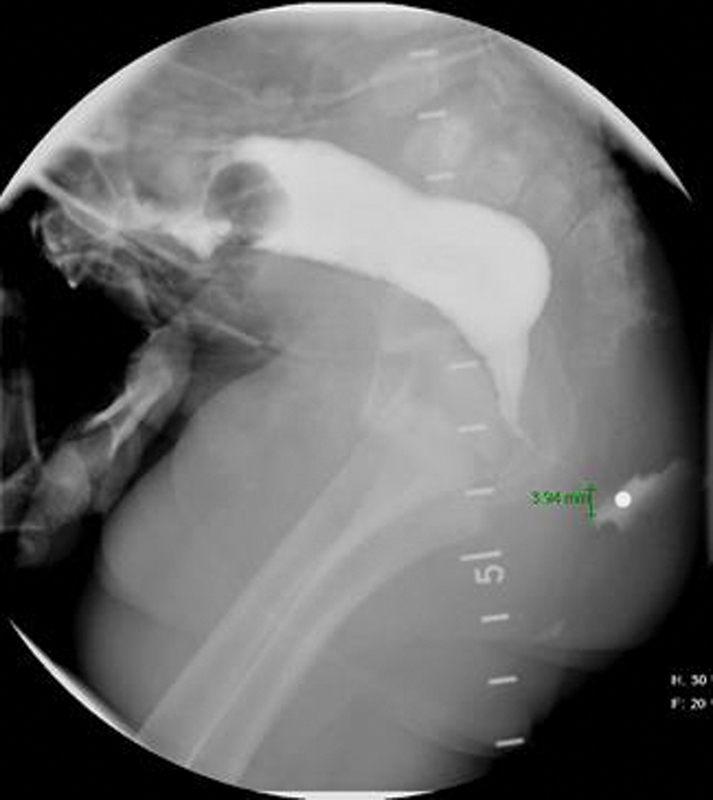
Distal colostogram demonstrating anatomy of anorectal malformation.

A 6-month-old male infant with an anorectal malformation (ARM) who underwent a colostomy as a newborn, now presents to your clinic for preoperative planning for his definitive repair. You review the distal colostogram ( Fig. 1 ) to assist you in identifying the nature of the malformation as well as to decide on the ideal surgical approach for the repair.

## Discussion

**Fig. 2:**
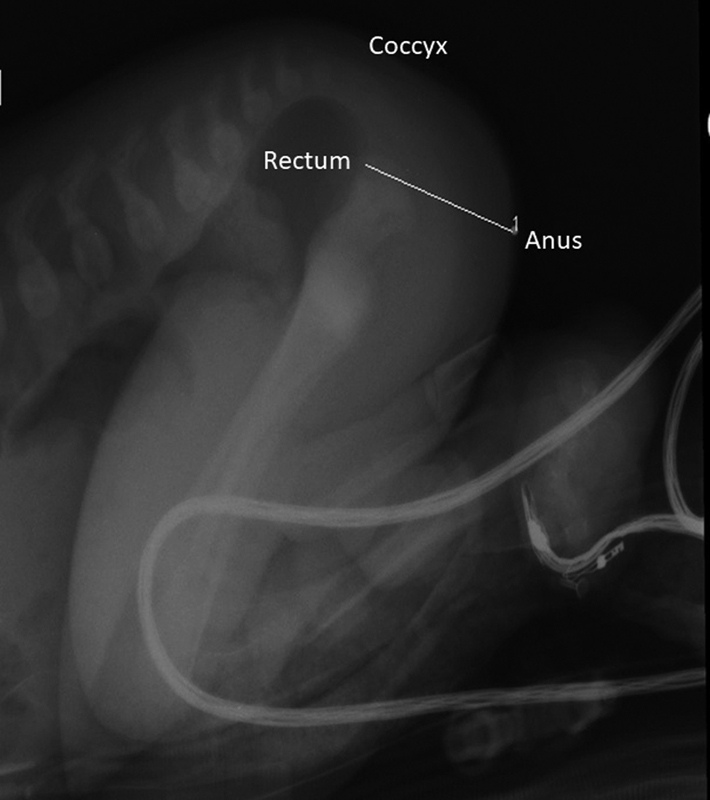
Cross-fire X-ray showing air column in rectum in relation to perineal skin and coccyx.

ARM is a common congenital condition that occurs in one out of 5,000 births and is slightly more prevalent in males. 1 The anatomic variants for ARM depend on the gender of the patient and the distal rectal anatomy. For males, the possibilities include rectal fistulae along the path of the urethra (bladder neck, prostatic, and bulbar), a no fistula defect, or a rectoperineal fistula. 1 During the newborn period, the likelihood of a rectourethral fistula and the predicted distance of the distal rectum from the perineal skin are both taken into consideration when contemplating creation of a diversion. In the case presented, there was no evidence of a perineal fistula in day 1 or two of life, and the air column in the rectum on a lateral prone X-ray was high above the coccyx and perineal skin, prompting the decision to create a colostomy ( Fig. 2 ). 2 Subsequently, when the distal colostogram was performed, it demonstrated the presence of a rectoperineal fistula with the rare situation of a long-narrow fistula ( Fig. 1 ). What is particularly noteworthy in this case is that if a rectoperineal fistula is evident at birth, a surgeon can usually reliably expect the normal caliber rectum to be within 1 to 2 cm of the perineal skin. In the patient presented here, this was not the case.

Most newborns with ARM first present with distal bowel obstruction and no anal opening on exam. 2 The newborn is observed during the first 24 hours of life for evidence of fistula, which is usually demonstrated by meconium excretion through the skin of the perineum or in the urine. 1 The former is indicative of a rectoperineal fistula, which can often proceed to a newborn repair via PSARP without the need for diversion. Any evidence of a rectourethral fistula warrants diversion with colostomy and delayed repair. 1 Not all fistulas can be visualized on exam, however, and further assessment is often necessary. If no fistula is apparent after 20 to 24 hours, a lateral prone X-ray (cross-fire) should be done to identify the presence of air in the rectum in relation to the coccyx and the perineal skin. 1 The presence of air in the rectum below the coccyx and within 1 to 2 cm of the perineal skin usually indicates that a repair via PSARP in the newborn period is possible. If air in the rectum is above the coccyx or greater than 2 cm from the perineal skin, then a diverting colostomy is recommended.

The guidelines explained above are based on the concept that when a surgeon approaches the rectum via a posterior sagittal incision, they must know where the rectum is expected to be found. If the surgeon can reliably determine that the rectum will be the first structure encountered, then a primary repair is safe. If, however, it is unclear where the rectum is, a blind perineal approach is fraught with danger, and a colostomy and subsequent distal colostogram are needed. A divided proximal sigmoid colostomy done in the newborn period allows for a safe path for stool clearance, a means to perform a distal colostogram to better understand the anatomy, and protection of the future anal repair.

**Fig. 3:**
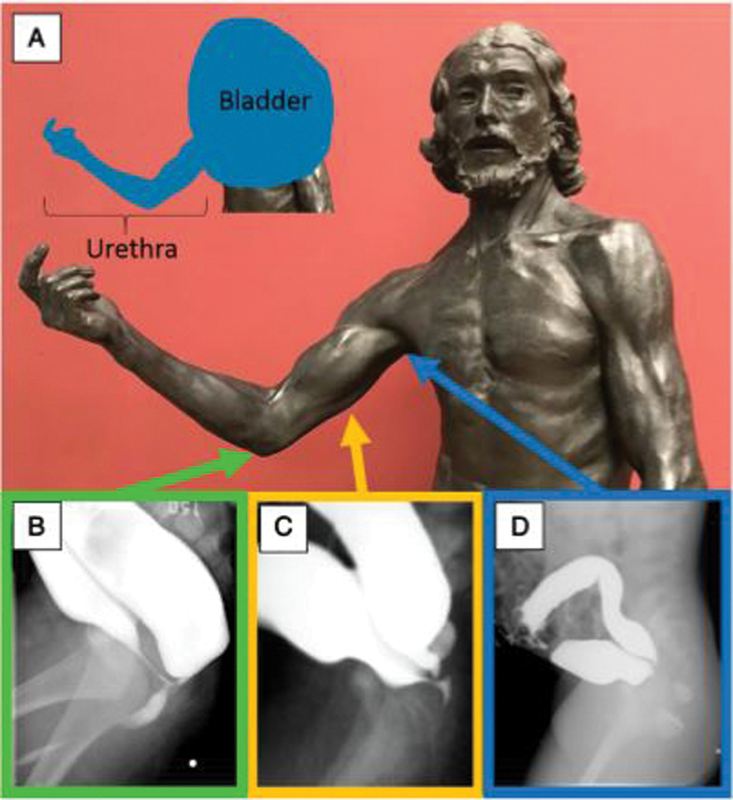
Auguste Rodin's Saint John the Baptist sculpture (1880) in the Musée d'Orsay, Paris, France. The right arm is used to represent the course of the male urethra ( **A** ). The relative positions of the male urethra are represented by the various levels of the arm of the statue. The elbow represents the bulbar urethra ( **B** ), the humerus represents the prostatic urethra ( **C** ), and the axilla represents the bladder neck ( **D** ). Reprinted with permission from Halleran et al. 4

To properly perform a distal colostogram, hydrostatic pressure is applied, which allows the contrast to overcome the striated muscle surrounding the rectum and to reveal the presence of a fistula. 3 This approach is useful for rectourethral fistulas, where fistulous connections may exist at varying locations along the genitourinary tract ( Fig. 3 ), 4 and for the rare case of a rectoperineal fistulas with long-narrow tracts such as the patient presented. In this patient's colostogram, the length of the fistula is approximately 1.2 cm ( Fig. 1 ). The rectum is reachable from a posterior sagittal incision, and laparoscopy or a transabdominal approach is not necessary. The initial diversion was correct as the air column was a significant distance away from the perineal skin, but usually a perineal fistula in the newborn period allows for a newborn repair without a colostomy. This case highlights a rare anatomic set-up of a rectoperineal fistula.

## Conclusion

In the male newborn with ARM, the likelihood of a rectourethral fistula and the predicted distance of the distal rectum from the perineum are both taken into consideration when contemplating creation of a diversion. The newborn with ARM with no rectoperineal fistula on examination in the first 24 hours should undergo a cross-fire film to assess the location of the distal rectum. Patients with evidence of a rectourethral fistulas or air 2 cm or higher from the perineal skin on the cross-fire film should undergo diverting colostomy. A subsequent distal colostogram plays a crucial role in identifying the exact anatomy of a patient with ARM and in determining the most appropriate surgical approach.

## Notes

New Insights and the Importance for the Pediatric Surgeon The newborn with anorectal malformation with no rectoperineal fistula on examination in the first 24 hours should undergo a cross-fire film to assess the location of the distal rectum. If the rectum is 2 cm or higher from the perineal skin or above the coccyx, a blind perineal approach is fraught with danger, and a colostomy and subsequent distal colostogram are needed.
